# Supplementary material for: Lack of pocket money impacts Ethiopian undergraduate health science students learning activities
Source: PLoS One. 2020 Dec 9;15(12):e0243634. doi: 10.1371/journal.pone.0243634 (PMC7725350; doi:10.1371/journal.pone.0243634)
Supplement: S1 Appendix — (DOCX) [file pone.0243634.s001.docx]

# **S1 Appendix: Information Leaflet for participants**

Title of Study: Lack of pocket money impacts on learning activities: Ethiopian undergraduate health science students

What is the purpose of the study?

The main purpose of this study is to investigate perceptions of undergraduate economically challenged undergraduate clinical year health science students on how lack of pocket money affects their learning activities. The study will involve interviewing financially challenged clinical year medical radiologic technologist students to enable a deeper understanding of their experiences as a student in Higher Education and during clinical years.

What will your participation involve?

If you are selected as one of the participants you will be asked to participate in an interview. The interview will consist of you telling me your experiences as a student studying in higher education and clinical placement learning. During the interview the role of the interviewer is to listen to your perceptions and experiences. There are no rights or wrong answers.

How long will the interview be?

The interview may last up to 20 minutes and will be held at department of radiologic technology department and a time convenient to you. With your permission tape record of the interview is taken as it will enable me to accurately record your experiences as it would not be possible for me to write the entire interview. However at times I may take some notes during the interview and you are free to look at these notes. It is my intention to interview you once, but I should be grateful if you would give me permission to return for a second discussion if it is necessary.

What happens to the information when it is collected?

When the interview is completed I will transcribe your interview onto paper exactly word for word. I then will carefully read your story and begin the process of looking at the information for themes and meanings. The tape recording and any subsequent printed transcripts of the interviews will be stored in a locked filing cabinet. Any information transferred to a computer will be password protected. Your name will not appear on the interview tape or the transcript. Each tape recording and printed transcript will be given a number for identification purposes.
