# Supplementary material for: Lack of pocket money impacts Ethiopian undergraduate health science students learning activities
Source: PLoS One. 2020 Dec 9;15(12):e0243634. doi: 10.1371/journal.pone.0243634 (PMC7725350; doi:10.1371/journal.pone.0243634)
Supplement: S2 Appendix — (DOCX) [file pone.0243634.s002.docx]

S2 Appendix: Semi-structured interview guide

# Addis Ababa University College of Health Sciences, Department of Health Science Education

Location: CHS-AAU department of radiologic technology

Date: _____________________

Time discussion starts: _____________ Time discussion ends: ______________

**Socio demographic characteristics of the participants**

Age: _____________________

Educational status: _____________________

The questions intended to explore the challenges that undergraduate health sciences students in their clinical year face with limited pocket money, as well as how students perceive these limited funds affecting their learning activities and their ability to meet challenges. The outcome of the study may help concerned body to recognize the impacts and give due attention to curb the problems posed by lack of pocket money.

All questions built up are intended for the academic purpose to alleviate problems due to students' financial challenges on learning teaching activities. Information obtained and your response will be kept confidential. Hence you are not required to tell your names during the discussion.

**Questions: Semi-structured interview Questions**

1. First of all, I am going to ask you some questions about yourself?
2. Now I want you to tell me some of your experiences in school.
3. Can you tell me about joining Addis Ababa University?
4. Do you think that your expenses increased in university?
5. Who are the sources of pocket money you used?
6. Is the pocket money you receive enough to cover your expenses?
7. Could you describe how the shortage of pocket money affects your learning activities?
8. Do you think that undergraduate student needs financial support
