# Supplementary material for: Lack of pocket money impacts Ethiopian undergraduate health science students learning activities
Source: PLoS One. 2020 Dec 9;15(12):e0243634. doi: 10.1371/journal.pone.0243634 (PMC7725350; doi:10.1371/journal.pone.0243634)
Supplement: S1 File — (DOCX) [file pone.0243634.s003.docx]

**Appendices**

**Appendix A: Information leaflet for participants.**

**Title of Study**: Exploring the effects of lack of pocket money on undergraduate clinical year health science students’ learning activities.

What is the purpose of the study?

The main purpose of this study is to investigate perceptions of undergraduate economically challenged undergraduate clinical year health science students on how lack of pocket money affects their learning activities. The study will involve interviewing financially challenged clinical year medical radiologic technologist students to enable a deeper understanding of their experiences as a student in Higher Education and during clinical years.

What will your participation involve?

If you are selected as one of the participants you will be asked to participate in an interview. The interview will consist of you telling me about your experiences as a student studying in higher education and clinical placement learning. During the interview, the role of the interviewer is to listen to your perceptions and experiences. There are no rights or wrong answers.

How long will the interview be?

The interview may last up to 20 minutes and will be held at the department of the radiologic technology department and time convenient to you. I will with your permission tape record the interview as it will enable me to accurately record your experiences as it would not be possible for me to write the entire interview. However, at times I may take some notes during the interview and you are free to look at these notes. It is my intention to interview you once, but I should be grateful if you would give me permission to return for a second discussion if it is necessary

**What happens to the information when it is collected?**

When the interview is completed I will transcribe your interview onto paper exactly word for word. I then will carefully read your story and begin the process of looking at the information for themes and meanings. The tape recording and any subsequently printed transcripts of the interviews will be stored in a locked filing cabinet. Any information transferred to a computer will be password protected. Your name will not appear on the interview tape or the transcript. Each tape recording and printed transcript will be given a number for identification purposes.

**Appendix B: Consent Form**

Addis Ababa University College of Health Sciences, Department of Health Science Education

The purpose of the study is to explore the effects of lack of pocket money on undergraduate clinical year health science students’ learning activities.

I agree to take part in the exploring of the effects of pocket money on economically challenged undergraduate clinical year health sciences students on their learning activities. I have read and understood the study purpose as described above. I understand that agreeing to take part means that I am willing to:

1. Agree to be involved in the semi-structured interview

2. Agree to allow the interview to be audio-taped

I understand that my participation is voluntary and that I can withdraw at any stage of the project without being penalized or disadvantaged in any way. I understand that any data that the researcher extracts from the focus group for use in reports or published findings will not, under any circumstances, contain names or identifying characteristics.

Participant’s name: ____________________________

Signature: _______________________________

Date: _________________________________________

**Appendix C: Opening**

Hello, my name is DebeaGela.I am going to have some discussions with you on the effects of lack of pocket money on undergraduate clinical year health science students’ learning activities, the case of medical radiologic technologists at Addis Ababa University.

Have you any questions?

Let’s go over some rules. First, let’s turn off our cell phones so we are not interrupted.

**Appendix D: Questions**

Addis Ababa University College of Health Sciences, Department of Health Science Education

Semi-structured interview guide.

Location: CHS-AAU department of radiologic technology

Date: _____________________

Time discussion starts: _____________ Time discussion ends: ______________

Socio demographic characteristics of the participants

Age: _____________________

Educational status: _____________________

The questions intended to explore the effects of lack of pocket money on undergraduate clinical year health science students’ learning activities. The outcome of the study may help concerned body to recognize the impacts and give due attention to curb the problems posed by lack of pocket money.

All questions built up are intended for the academic purpose to alleviate problems due to students' financial challenges on learning teaching activities. Information obtained and your response will be kept confidential. Hence you are not required to tell your names during the discussion.

Questions: Semi-structured interview Questions

1. First of all, I am going to ask you some questions about yourself?
2. Now I want you to tell me some of your experiences in school.
3. Can you tell me about joining Addis Ababa University?
4. Do you think that your expenses increased in university?
5. Who are the sources of pocket money you used?
6. Is the pocket money you receive enough to cover your expenses?
7. Could you describe how the shortage of pocket money affects your learning activities?
8. Do you think that undergraduate student needs financial support?
