# Supplementary material for: Lack of pocket money impacts Ethiopian undergraduate health science students learning activities
Source: PLoS One. 2020 Dec 9;15(12):e0243634. doi: 10.1371/journal.pone.0243634 (PMC7725350; doi:10.1371/journal.pone.0243634)
Supplement: S1 Coding — (DOCX) [file pone.0243634.s004.docx]

**Semi-Structured Interview Coding**

| **participant** | **Transcripts** | **Codes** |
| --- | --- | --- |
| A | I think my expenses in the university totally increased. Let me give you one example; when we were with our parents' breakfast, lunch and dinner is covered by parents. | Cost of attending university  Timing of access to food at home |
|  | And Here in the university food is prepared by the university and given at a specified time and once you missed the time to eat we enforced have to buy the food. Thus this increased the expenses. | Timing of having dinner at university  Missing food time at university  Enforced to buy food |
|  | The other thing is you have to pay for copying handouts. | Expenses of for copying |
|  | The time changes fast. For example, the power of purchasing of our birr when we were in year one and two are not the same compared with when I was in year three and four. | Inflation  Inflation  Reduced power of purchasing of birr |
|  | In year one and two we remain in the university campus and no more expenses other than for food, copying and other stationary materials. However, in year three when we go for practice the expenses definitely increased. | Lack of transportation  Coming late to class  Missing class  Cost of food  Printing |
|  | My families support me financially as much as they can. I have searched if there are financial aids in the university, there are very limited ones. I got scholarship from Aklilu Lema foundation. | Sources of pocket money |
|  | Let me explain my own experience. I got Aklilu Lema foundation scholarship. It gives scholarships based on your extra-curricular activities and academic achievement and not financial aid for needy There is a difference before getting Aklilu Lema foundation financial aid and after getting it. I cover expenses which cannot cover by money from families. | Getting financial aid relieve financial status  Aklilu lemma scholarship improved my financial status |
|  | Let me tell you the truth. If I want to have what is necessary for me the money that I get is not enough. But, I have to compromise some needs to survive. | Insufficient pocket money  Limiting needs  Restricted daily activities  Compromising some of needs |
|  | Okay! Lack of enough pocket money affects my daily activities. It also affects academic activities to some extent. | Lack of pocket money and learning  Insufficient pocket money  Source of Food  No money and limited daily activities  No money and reduced learning activities |
|  | I cannot say a single specific challenge here. For example when I run out of pocket money I go on foot. Due to this I sometimes come late to the class and this makes me not to attend the class attentively. And other time I miss class. | Sacrifices when unable to afford transportation cost  unable to afford transportation to class  missing class  coming late to the class  loss of attention in class |
|  | Yea. My expenses in the university varied during different years of study. For us when we start clinical practice the cost of transport definitely increased. | Low Pre-clinical year's expenses  Increased expenses in Clinical years  Clinical placement expenses  Expenses and year of study |
|  | And the way that the universities provides us transportation service and provide the cost of transport is not as such comfortable that we enforced to use taxi and pay out of our pocket. The tariff of transport varies; sometimes it changes every fifteen days. | Transport cost changes infrequently  Money from parents is not enough |
|  | I do not believe that the problems get attention. However those who are administer try to find solutions. But we see no solution yet. | No attention from officials |
|  | Okay! I think teachers understand students' problem to some extent. For example, when students come late to the class they allow us to enter to the class. | instructors understand the challenges  food preferences  timing of paying cash money for non-dormitory and non-cafeterias  amount of money paid for non-dormitory students |
|  | **yea.** I think it better if more research is done on this area. How much students need for their expenses? Do the universities ready to provide students with pocket money? Do the existing financial support giving enough pocket money or not? So the first step is to identify the problem and know it. Then providing students with pocket money will curb the problem. | The problem seek great attention  Some changes must be incorporated in cost sharing system |
|  | The other think is the university gives all of the services to the students in kind. I think it is better if some services in cash added to the cost sharing. |  |
|  | When you are getting in-kind services, it is connected to cafeteria and dormitory services. The quality of food is not so good. And those who get cash are so in problem. The money is not paid on time. Sometimes they give after five months and the amount of that money is not enough. | Improving services |
| B | Even though my parents are not educated; they know the advantage of the education. There is understanding that education gives respect in our village; thus joining university is seen as a great achievement. That is it | Poor Parents' understanding about expenses in the university |
|  | Okay! My Parents take as the government covers everything. And they assume that they are not more expenses. We use exercise book to write on when we were with our parents in lower schools and high school. Parents do not know about handouts, assignments and that go for practices using transportation. They think that the government feed them and they are no more expenses. So as I have told you they are not educated that they did not experienced. Therefore, they do not know that they are more expenses. So there are many problems | Problems raised from parental misunderstanding |
|  | You have to manage everything. Here there is no family you guide you. There are so many students. Some smokes and some drink alcohols. It is you who decide your future. | Self-management  Peer influence  Self-control |
|  | Now I spend on are mobile cards and here the cafeteria opened at 4:30 pm. So eating at that time and studying in the night is difficult. Since studying needs more energy you have to eat. So most of my expenses go for these spends: mobile card and food. | Cost of mobile card  Too early Dinner /inappropriate dinner time  Difficult to study in the night  Spending on additional foods  Need more energy to study |
|  | I get the money that I use from my parents and I do not spend for unnecessary things. When I run out of pocket money I borrow from my friends and give them back when money comes from my parents. | Effective use of pocket money  Culture of borrowing |
|  | No one sends me; my close families assume that my parents are enough for me. However, when I go there some give me for tea; but they do not think that I face so many challenges. | No support from extended families |
|  | It may be enough if I only use it for only most important things effectively. The most important things are like buying soap, for transportations that you have plan carefully. However, if I want to eat every night and want to live as my friends it is not enough. | Pocket money utilization  Prioritizing expenses  Enforced to stay hungry  Great differences among classmates |
|  | As We live in Addis Ababa. My friends usually come to dorm buying fruits and they go and eat what they want; so I cannot get these and the likes of these. | Impact from the place of university  Great differences among friends |
|  | I cannot go with friends to get refreshments. | Lack of refreshment |
|  | I cannot assess materials on internet in my dorm; as my balance does not allow me. So I have to find Wi-Fi which is not available at safere selam campus. So I have to wait till I come to black lion campus to download learning materials from internet. Look if I have enough money I can full my mobile balance and access on my phone at any time and place. Therefore, having not access to to internet at time I want; this affects my learning. | Unable to use internet on phone  Lack of free wi-fi at Sefere Selam campus  Different educational facilities at two campuses  No access to internet affects learning |
|  | I do not think the university administration bodies understood the problems students faced due to financial restrictions. If they understand it they have asked us. If they now, they may went around and asked us. But no one did it. In my opinion if they understood it they may try to solve it. | Challenges are not get attentions |
|  | Okay! I think the university from the top must form committee who do these work. And students 'may be students association must go ask the university about the situations. And teachers must approach the students and understand the status of their students and have to support them. | Special office must be established |
| C | My families have good understandings. They are informed about higher education | Good family understanding of higher education |
|  | Here in the university I need a lot of things which I must spend for it. For example when I was with my parents food, soap and everything I need provided by them. Here I need a lot. I need money for buying soap, for taxi, for stationary materials, and I need money for go outside campus for refreshment with my friends. | Basic needs  Transportation cost  Cost of stationary materials  Cost of refreshment |
|  | My parents send me for my expenses, and my brother and uncle send me some money. | Sources of pocket |
|  | It differs from person to person. For example I use cafeteria. But since, the food is not so delicious I sometimes eat outside the campus | Cost of additional food |
|  | Okay! For me not having enough money matters. For example without eating well you cannot learn as you want. Look when I hungry I cannot read. I get tired and sleep. | Enforced Stay hungry  Challenges from lack of money  Food and study |
|  | I do not believe that university officials understand the challenges. For example Addis Ababa University has a lot of campuses. When we go to main campus the system is different. When we go to sefere selam there is no internet access and the electricity is not so good. | Different campus facilities |
|  | Here in Tikur Anbessa Wi-Fi is available but no wi-fi access at sefere Selam where we live. | Different campus with different educational facilities |
| D | My parents are not educated. However, my aunts and uncles are educated to university level. They have positive understanding of higher education even though they are not educated. | Parents and higher education status  Close families' understanding of higher education |
|  | Okay! From my parent side there is thinking that there are no expenses in the public university and my opinion was the same. I have the information that there will be no printed books given like in high schools. Therefore, I did not have any idea other than expenses for books. It is talked that everything will be covered by the government. Thus, I never thought that there are more expenses in the university. However, after coming here it is beyond my expectations. It is very expensive. | Parents and higher education  Self-management  Basic needs  University expenses |
|  | After coming here while I was freshman and after that more we face that more expenses are expected. Look we live away from our families and even being it Addis Ababa; moving from place to place even going to church require transportation (taxi).so you pay for it. And the other thing is food. When I was a freshman the food are not so comfortable; that I spend money for it. Being with classmate: there are costs .I cannot explain how much I need and I have to spend. | Expenses in the higher education  Being in capital city increased expenses  Food expenses  Food preferences  Cost of refreshments |
|  | I get money from a lot of individuals even though the family covers most of it. I have relatives here who help me and also there are relatives who live abroad who support me. So they cover it. Here, in Addis Ababa I have aunt and uncle. And recently my brother started job here in 2018. And one of my uncle lives in America. Therefore, I get small money from each of them. | Multiple sources of money |
|  | I never think that the pocket money it is enough for my daily expenses. | Insufficient pocket money |
|  | There are a lot of problems which I faced. For example, in our department when we go for clinical practice site there is no money provided for transport. There was no transport service. I remember that I go on foot for many days to clinical practice. Because I had no money in my pocket and there was no transport service, but there was attendance at the clinical practice area; therefore the only choice I had was to go on foot. In addition to this I was sick for some time and I could not able to use the cafeteria and there was no money in my pocket and I was enforced to borrow from my friends. This is common in university; students borrow money from one another. Therefore, we share with each other. | Problems due to lack of pocket money  Transportation cost  Sacrifices due to lack of pocket money  Poor food quality  Expenses for food  Lack of pocket money  Food and study |
|  | Look during the first year I was so confused on what you spend. Now we can manage on what we spend. We advanced in years the expenses increased. One is the cost Transportation for clinical practice. Look in the final year you need more expenses for graduation. | Increased expenses with years of study  Clinical placement transportation cost |
|  | The universities know more than enough. But I do not think the university even tries to give solutions to these problems. They always informed about the problems, but never try to find solutions. That means the university is not willing to solve the problems. Starting from the lower administration to the higher they are not even happier when they asked for some solutions. For example when they asked transportation service for clinical practice they are not willing to answer; they say we are not given instruction from above body. And they never want to listen to the problems.  Instructors cannot avoid their problems as they do not have power. I think they are happy if the problems are solved. Look at department level the power is restricted. Some even give us to cover cost of the transportations. | Instructors support |
|  | These problems are chronic problems. It is not new problem. All stake holders must give attention to solve it. Some problems may not reach the higher university officials. |  |
| E | Even though they did not attend the university they have good understandings of it since they had attended college | Parental positive understanding of higher education |
|  | There are so many challenges as we are away from your families; the way you manage yourself to live with different classmates of personalities. | Self-management |
|  | University is a place where great life challenge is. It is a place where you face a cross-road: way to bad and good. So to withstand theses challenges you have to control yourself. Over all there are a lot of challenges. However, it depends on ones strength; you have to control yourself. | Challenges in university  Self-control |
|  | The first thing is separated from from families and you are enforced to live with different persons of different behavior. You have to cope up with them | Separation from families  Different environment |
|  | The other, is everything you use come out of your pocket. You cannot ask your families daily. You are giving money for a period of time and use from that money for little and big things. Therefore, this is one challenge. | Need of pocket money  Not enough money |
|  | In general, using the money provided to you effectively is so difficult and the education is also somewhat difficult. | Money management  Educational activities |
|  | In our daily activities starting from the very smaller things like payment to buy pen, notebook, copying handouts, printings and in our campus there is no good internet connectivity sometimes we go out and use internets by paying for it. And since, Addis Ababa is a capital city we move by taxi. In general, the expenses are beyond our expectations. | Every dayd expenses  Internet connection  Unexpected expenses |
|  | Of course it is different. Because, the learning-teaching method and the environment where you stay differs from from pre-clinical year and clinical year what I was experienced. For example in our case during first year there is no place we go. We stay in the campus and learn there. And in the second year we start clinical practice. The service (transportation which is provided by the government) is not as we think. Therefore, we are enforced to pay for transportation. Therefore, the expenses vary from year to year. | Clinical year uniquity  Transportation costs |
|  | I have no sources of pocket money except from my parents. | Sources of pocket money |
|  | I did not get any financial aid yet. However, I have applied to Aklilu Lema foundation and won it. But, I did not get the payment yet. However, Aklilu Lema foundation is not a financial aid to the financial challenged students. It is a merit based financial support. Every Addis Ababa university student apply based on their academic achievements. So it is like award not support. | No financial support |
|  | Okay! If I want to spend it for everything I was it is not enough. Look when you live in the campus, there are a lot of problem. I do know if it is poor management or poor performance; you spend unnecessary expenses. Family thinks that my son uses the cafeteria and dormitory; and no need of money for food. So they send the money for tea and other expenses. However, here due to poor administration students exposed to unwanted expenses. For example, the campus where we live and the campus where we learn are too far from each other. Transport do not come when we need and when available transportation is not so enough for the students. They often send us only one bus for so many students. Due to this we usually use taxi. And it is the same when we go for clinical practice areas. In addition, the most critical one is cafeteria; we do not know if the government knows it or not; the food prepared in the cafeteria is not the kind of food you always eat. You eat it selectively. Therefore, sometimes we enforced to eat out side which increases our expenses. | Insufficient pocket money  Pocket money management  Different campuses for learning  Transportation cost  Uncomfortable food  Food expenses |
|  | It is difficult mention the problems imposed on me due to lack of pocket money. It limits you so that you cannot move as you want. Because, you need to save your money for the most important things and limit your wants. | Challenges due to lack of money |
|  | I do not think they understand it. Because if they understand it they do not increase our expenses while they are expected to decrease it. I do not know if is purposely or unknowingly that they are carelessness affects us. For example, the university has enough cars and car drivers. However, those who take the responsibility of monitoring the the student transportations have a huge problem. Thus, due to their carelessness we are exposed to extra expenses. | Poor university management |
|  | When there is the problem of transportation instructors' help in rearranging the class but not financially. Thus, this is somewhat better than those who are at the managing and administration. In addition, instructors limit the handouts which require printing. This reduces our costs. | Instructous understanding of situations |
|  | In my opinion if those at managing and leading position deliver their responsibility effectively they will reduce many student expenses and other challenges. For example, if those who are working related to the cafeteria effectively do their responsibility the student may not expose to unnecessary expenses. If the transportation improved the expenses will totally reduced. The other thing is it is difficult to identify the needy student here. If you are at high school students who need extra help are known and supported. I think no attention is given here in the university. |  |
| F | My parents have their own business; they work in market. They learned up to seven grades each. They do not have enough understanding about university. They do not care about education. You have to learn on your own. | Family educational background |
|  | I come from home to learn. The university gives money in cash for dormitory and cafeteria. And this is not always paid on time. For example today we are around the end of January and I am not paid for it. So the governments do not cover your expenses.  As I said the government does not cover everything you need. When there is class I come from home covering the taxation and staying here, I spend for food and others. So the expenses increased |  |
|  | Is better if to explain the intensity of this in terms of days. For me when I status full day my expenses increased and if I come for have day it totally decreased. The other things, when I was fresh he in year one I do not spend much money. |  |
|  | The money I get is not enough, but I have to manage it to my urgent and important needs. If I spend what I get on what I need, I will get in to financial crisis. That is it. |  |
|  | The university officials do not understand. |  |
|  | Look, my families may think that she has every stationary material she need and has no problem you know; when you are educated you learned something. Our community lacks this. And the other things is sharing resources is so good if the common understanding is created. Look when you consider university one come having lunch the other without out eating; there are so many differences. We have to share our resources to alleviate these and the likes of problems. |  |
| G | It is not as I think it is difficult. When you are with family you do not think a lot. Here you have to mange yourself. And I think the education is difficult, but even I come here if you study hard is very simple. You can succeed |  |
|  | Here you have to make a good relationship with students if you want to succeed. |  |
|  | : I only think that I have spent for additional food only. But it is not. Here; there is cost of transportations and others which you do not expect. For example, you can go with your friends. Look the mobile card you use increased here. You have to always fill your mobile and call classmates for information every time. |  |
|  | The money I get is not enough for my important expenses.  My expenses increased when you go for clinical practices. |  |
|  | When I ran out of money. I cannot go for clinical practices. Due to this I miss cases which I learn from day to day as there are different cases which we encountered on different days. And if I am at sefere selam and I missed the bus I am enforced to be absent from the class. Missing class I miss a lot from teachers. |  |
| H | My parents believe that we can be changed by education. However, they do not have and they cannot provide us better things. That is it. | Parental understanding  Parental financial status |
|  | My expenses increased after I join Addis Ababa University. Before I join here I eat food from the home. There were no expenses for the food. | University expences |
|  | Okay! My parents give me little from what they have. Other than this I cannot get any money. I try to get money by doing different things.  I got 500 birr monthly. 400 hundred birr is even not enough to cover the launch. Look when you come together with your friends you need coffee-tea ceremony. You are young boy; you need to make relationship. It is not enough. | Income  Trying to get money  monthly income |
|  | When I failed to get what I what you get depressed. You become depressed. For example, when your friends go to cinema and you failed to do nothing I feel it. | depression |
|  | Look it affects me in different ways. Look the depression affects your learning. And sometimes if you do not eat you get hungry. And you cannot study. Some times when I placed far for clinical learning and I did not have transportation money that I was in trouble. This affects my learning. Look, I only fill my mobile balance very infrequently. When you fail to use power points it is very hard to follow my education. | Impacts of lack of pocket money |
|  | The expenses vary a lot among different years of study. When we advance from year to year our expenses increased increase. There is also inflation. |  |
|  | For example, when I go for clinical learning. When I came from it if I cannot afford for lunch I go the class without eating something. The class you attend while hungering is nothing; you may not think about the learning which takes place. |  |
|  | I do not think instructors understand everyone's situations. I think some teachers understand and some never understand. When you come to class late some and told the case some did not understand and enforce me to go out of the class. I miss quiz. This affects my learning. |  |
| I | My family believes that once you enter university everything you need is provided by the government. They assume that you only need money from them for soaps and other smaller things. They have like these understandings. | Perceptions of university expences |
|  | I think my expenses increased. Look when before I came here, I have been with my local friends and I see no great difference in clothing; here students came from urban areas and I sometimes compare myself with them. | University expenses |
|  | My parents and my older sister support me. MY uncle sometimes supports me a very little money | Sources of money |
|  | I get about 500 birr per month. | Monthly income |
|  | This is not enough. You have to study for longer time; so you need to eat. And there are a lot of other things to spend on. | Insufficient money |
|  | Some Teachers understand students' situations |  |
|  | One has to use what we get properly. Second you have to convince your family and others who you think help about the expenses. The other is you have to ask for financial aid. | Pocket money management |
|  | I think the university must understand the situation of where students come. Look in our department the cost for transport when we go for clinical placemat did not paid on time and if you have no pocket money you get into trouble. They have no idea how student go for practice. So they have to give solutions. | Clinical practice costs |
| J | University life is not as I thought before; there are few difficulties. When I depart from my family and start to live on my own it is difficulties have to think about a lot of things. |  |
|  | When one think about university it is better to have a good relationship with other students. You have to make a good relationship. | Relationship and university |
|  | I had information that we have to pay for purchasing additional food. However, here there are a lot of things we spend for. There is a cost for transportation and others like mobile card. | Cost of food  Cost of Mobile card |
|  | Look I may celebrate a happy birth days with my friends. There is mobile card. And during clinical years we spend for transportation. | Entrtainment  trasportation |
|  | When I run out of money I could not go even for clinical practices and when I am at sefere selam and if I missed a bus then I miss class from black lion. I miss cases from clinical practice and miss a lot from lecturers. That is it. | Pocket money and clinical practices |
