# Supplementary material for: Lack of pocket money impacts Ethiopian undergraduate health science students learning activities
Source: PLoS One. 2020 Dec 9;15(12):e0243634. doi: 10.1371/journal.pone.0243634 (PMC7725350; doi:10.1371/journal.pone.0243634)
Supplement: Attachment — Submitted filename: Response to Reviewers.docx [file pone.0243634.s005.docx]

To:	Richard Bruce Mink, Academic Editor

PLOS ONE

Subject: Point-by-point response to academic editor and the reviewers’ comments

First of all I would like to offer my sincere gratitude to academic editor and the reviewers of the at PLOS ONE journal. We made the changes in green color. The manuscript is also edited by professional editor and changes made submitted separately.

We modified comments point by point and submitted accordingly.

Response to Academic Editor

Please ensure that your manuscript meets PLOS ONE's style requirements, including those for file naming.

*We have changed the manuscript using PLOS ONE style guideline. Files are also named accordingly.*

Please modify the title to ensure that it is meeting PLOS’ guidelines.

*We modified the title to: Lack of pocket money impacts on learning activities: Ethiopian undergraduate health science students*

*And added short title as; Lack of pocket money and learning activities*

Please justify the small sample size involved in this study with reference to other similar works.

*For our study we used qualitative study and to get the predefined objectives of the study we applied purposive sampling technique. During our data collection the data show saturation for what we aimed. Therefore, among twelve participants ten participants have been included in the study.*

Please ensure you have thoroughly discussed the potential limitations of this study within the Discussion section.

*We have added and discussed thoroughly the potential limitations of this study within discussion section*.

Please provide additional details regarding participant consent. In the ethics statement in the Methods and online submission information, please ensure that you have specified what type of consent you obtained (for instance, written or verbal, and if verbal, how it was documented and witnessed).

*We clarified the type of consent we obtained in the method and ethics statement.*

We suggest you thoroughly copyedit your manuscript for language usage, spelling, and grammar. If you do not know anyone who can help you do this, you may wish to consider employing a professional scientific editing service.

*We have revised the manuscript several times and look for grammatical errors, spelling errors and language usage.*

*The Corresponding author has communicated with Excision Editing; professional scientific editing service based in Canada and incorporated the changes from authors’ revision and professional editors.*

*Address of Professional scientific editing service*: [www.excisionediting.com](http://www.excisionediting.com)

*Changes made by professional scientific editing service are uploaded separately and changes are highlighted with red color.*

Please amend either the title on the online submission form (via Edit Submission) or the title in the manuscript so that they are identical.

*We made the title in the manuscript and online similar.*

Please include captions for your Supporting Information files at the end of your manuscript, and update any in-text citations to match accordingly.

*We have added Supporting files at the end of the manuscript and updated in-text citation.*

Response to Reviewer #1:

The prose can be edited for clarity. Also edit for grammatical and typographical issues.

- *The authors have revised the manuscript several times for grammatical errors, language usage and typographical issues and incorporated all changes. As well professional scientific editing service (Excision editing) has edited the manuscript. All changes are included in the revised version of the manuscript.*

The conclusion has to be supported by robust data. Consider amending the conclusions to suit the amount of data you have. Eg.To ask the government to adjust certain policies based on interviews of 10 students may not be justified.

- *We have revised our conclusion of the study. Data presented in the study used to dram conclusion. Example, we added these findings indicate that the department, the school, the college, and the university need to find a way to help students afford university, outside of their room and board. Moreover, creating awareness about student expenses at university within the community will likely improve students’ financial status. Additionally, teaching students self-management skills is also another area that could increase student success. Future studies should further investigate the factors that contribute to student financial hardship in the health sciences.*

Add in some benchmark information such as the monthly income of Ethiopians, the school fees, cost of transport etc, so that international readers can understand the context of figures you cited (eg. what is the value 500 Ethiopian Birr).

- *The column indicating average monthly income of students is deleted as there is no established benchmark.*

A more in-depth discussion on the significance of each of the Themes and Categories should be done.

- *The result has been modified significantly. We have thoroughly discussed each themes, sub-themes and categories.*

Consider making a Figure or Diagram to depict your findings graphically

- *The importance of depicting the findings graphically is considered and we developed a conceptual frame work. The conceptual frame work is added as Figure 1 in the result section.*

Consider clarifying the recruitment process of your interviewees in terms of anonymity, voluntary participation, why they need to be rewarded with mobile cards and money and whether they may be disadvantaged in any way should they not participate. This is important as they may be considered a vulnerable population.

- *Participant recruitment process discussed thoroughly in the method section. The rewards are not communicated before the interview and to give after interview is decided by authors. Each participant was informed during consent that participant or not participating will not affect them.*

Consider increasing the sample size, and also quantifying the challenges that they face in terms of how many respondents face each of the challenges you raised, and also rank these challenges in terms of importance to them. This can be done in subsequent studies.

- *The small size is considered as limitation of this study and we consider other future study.*

Response Reviewer #2

Background- should include a sentence on previous data and the gap in knowledge	We added the established data.

- *The cost of university presents various challenges with regards to students’ daily learning activities. This is particularly evident in developing countries, where higher education students face acute financial problems that greatly affect their daily educational activities. And gap of study in Ethiopian context is added as: health sciences students have additional costs during their clinical placements that are above the common expenses for university students.*

Objectives- tightened up to be more specific

- *Objectives are modified as: Authors aim to explore the challenges that undergraduate health sciences students in their clinical year face with limited pocket money, as well as how students perceive these limited funds affecting their learning activities and their ability to meet challenges.*

Methods- should start with we conducted 10 semi-structured interviews of a description of subjects, time course, and place.

- *The place and time of study specified in the method. As well the number of participants and tool of data collection discussed.*

Conclusion- should start with the conclusion and then advance to potential solutions. The solutions in abstract should have been discussed in the paper (e.g. educating students on money management)

- *We modified the conclusion section and added: Undergraduate students faced many challenges due to the lack of pocket money. Due to challenges students faced affects students learning activities either directly or indirectly. Additionally, teaching students self-management skills is also another area that could increase student success*

Should include previous data and studies on the challenges that students face and areas of potential misunderstanding (if documented) and then discuss the gap in knowledge.

- *Previous studies on students of low socioeconomic status and the international data have been assessed and the Ethiopian context was analyzed. The absence of study on the matter in Ethiopia is taken as a gap.*

Lines 68-70; is there a reference

- *Lines 68-68 are omitted.*

Lines 70-71; please explain what cost-sharing is

- *Cost sharing in Ethiopia is defined. Ethiopian Higher Education Proclamation and the Cost Sharing Regulations: provide room and board, and covers the tuition fees for those who students who pass Ethiopian Higher education entrance examination and joined public universities, however it does not provide students with pocket money for other expenses.*

Line 72; the cited article does not address pocket money

- *The referenced article is corrected to: Bonke J. Do Danish children and young people receive pocket money ? Rockwool Foundat Res Unit. 2013;(57).*

Line 75; the population belongs in the methods

- *Line 75 is omitted*.

Method: This requires major revision and clearly delineated sections- such as Participants/Recruitment/Ethical considerations, Interviews and then data analysis.

- *The method section is revised several times. And subsections are also well organized and enriched. Participants- who was eligible, Recruitment- who were they recruited; Participants selected using purposive sampling technique. Students who show financial support and submitting letter to the department in order to get financial support and those who declare themselves as financially challenged students were eligible.*

Lines 96-96-98 belong in results

- *Lines 96-98 have been modified.*

How was interviewer selected? The information about languages used should be in results, as also interview length.

- *The interviewer, Debela Gela, was an instructor from the Department of Nursing, AAU. The interviewer had never taught the participants, nor had any role in the Medical Radiologic program, and was fluent in Amharic and Afaan Oromo.*

Data analysis- what technology was used for transcription?

- *Authors transcribe manually. Three of the authors transcribed each interview individually and then finally come to consensus after several discussions over transcripts*.

Delete lines 115-116 because redundant.

- *Lines 11-116 are deleted*.

How did the group come to consensus on codes?

- *As we with transcription, authors code separately and then came together to discuss and combine the codes. Through several discussions consensus has been reached.*

Was saturation achieved?

- *Based on our objective the data became saturated*.

Table 2 should be under results- with elimination of column that includes codes. Headings should be domain, theme, representative quotations.

- *Table two moved to under result section.*
- *The column containing codes is deleted.*

Consider substituting

"Lack of family resources" for "Poverty"

"Difficulty asking for aid," for "poor skills of asking money

"Transportation" for "transportation expenses"

"Food" for "Food expenses"

- *We have substituted accordingly*.

It is unclear how the theme of "self-control" or "social life and learning activities" relate to financial limitations

- *Participants of the study stated that their lack of pocket money affected their ability to make connections with peers and noted that the lack of a social life affected their* learning activities. A few participants indicated that meeting with other students and understanding each other’s financial status is very helpful in creating a support network. Therefore, a student’s inability to go to the cinema, and/or attend ceremonies and other celebrations with their peers often led to students feeling depressed, which negatively affected their learning activities.

The segment summarizing the themes 127-131 belong in the results

- *The section is moved* into result section

Lines 132-135 are redundant and should be deleted

- *Lines 132-135 are deleted*.

The section on theoretical approach 136-154 should be deleted

- *The theoretical approach section is removed*.

Results: Start with the themes and domains that emerged

- *The result section is modified significantly. It is started with major themes and explained. And diagram depicting the findings is developed*.

The theme's and subcategories would be better served by capitalizing domains (themes) and using italics for "subcategories" or themes. Because the quotes are already included on the table which will be moved to this section, no need to include again in the narrative. Themes need further detail and elaboration; they are unclear.

- *Themes, sub-themes and categories are edited accordingly. And quotes are no longer used in the narratives.*

Discussion-

The connection to findings and Bourdieu's theory need further clarification. It is difficult to understand. Concepts are introduced in the discussions which were not described in the results (e.g. lines 337-341)

- *The discussion section has been modified and enriched. It is edited for misunderstandings and more clarified*.

Conclusion-

It is unclear how authors concluded that lack of funds adversely impacted clinical skill acquisition. Please specify how the Ministry of Science and Higher Education should revise the Ethiopian cost-sharing system.

- *Our data show lack of pocket money affects student’s class attendance, clinical placement attendance, and prolonged studying. This believed that students, miss learning different cases on daily basis as they arrive late, stay there while hungering and total absent. Based on participants’ ideas, The Ethiopian cost-sharing policy should be revised to allow for loans or scholarships to students, as well as paid clinical year internships for undergraduate health sciences students. Addressing student financial hardship, especially during clinical placements, is essential for improving the student experience and increasing student competency*.
