# Supplementary material for: Lack of pocket money impacts Ethiopian undergraduate health science students learning activities
Source: PLoS One. 2020 Dec 9;15(12):e0243634. doi: 10.1371/journal.pone.0243634 (PMC7725350; doi:10.1371/journal.pone.0243634)
Supplement: Attachment — Submitted filename: Rebuttal letter.docx [file pone.0243634.s006.docx]

Rebuttal letter

**To academic editor:**

Figure 1. There are four themes emerged from the data. In the conceptual framework we tried to show the relationship between limited pocket money and learning activities. Some of the themes emerged are factors leading to limited pocket money, some are emerged due to lack of pocket money and others are voce-versa. For instance, poor understanding of university expenses, lack of family resources and difficulty of asking for aid are the main factors causing lack of pocket money. While, maintaining social life and self-management could lead to limited pocket money and limited pocket money also lead to poor social life activities and poor self-management. On the other hand, limited pocket money leads unable to afford very important needs. Finally, the figure shows that limited pocket money and inability of affording very important needs Impacts on Learning Activities. Learning activities are affected by missing class, coming late to class, coming late to clinical, placement, missing clinical placement and loosing attention.

We correct punctuation whenever however is used.

**To reviewer 2**

- The central circle was pocket money and changed to limited pocket money. The directions show the relationship between limited pocket money, emerged themes and its impacts on learning. In some the limited pocket money has unilateral direction and in others bilateral and vice-versa.
- Table 1 and 2 are modified so that the participants’ orders are listed alphabetically in both tables.
- The discussion part is modified accordingly. The main findings are emphasized. And the implications and future steps are capitalized in the conclusion.
- We found the suggestion for improvement of theme titles and modified accordingly.
- Page 15, line 171 is changed accordingly
- Page 6, lines 107-108 is modified to thematic saturation is reached after reviewing the transcripts for ten participants.
- The first paragraph of the discussion part is modified. We tried to focus on the main findings in discussion followed.
- The conclusion summarized with implications and future steps in solving the raised in the paper.
- Referencing (table 2) redundantly in subthemes is correct, so table 2 is referenced in the result section and main themes.
